# The western transmission of traditional Chinese medicine: an investigation of the cultural elements of traditional Chinese medicine in biomedical systems of cross-Asia countries

**DOI:** 10.3389/fphar.2025.1589275

**Published:** 2025-07-07

**Authors:** Aibibula Kadier, Mihray Ablimit, Hebibulla Tursun, Nuramatjan Ablat

**Affiliations:** ^1^ College of Marxism, Xinjiang Normal University, Urumqi, China; ^2^ Xinjiang Uygur Autonomous Region Shache County Dunbag Township Health Center, Xinjiang, China; ^3^ Department of History, Peking University, Beijing, China; ^4^ School of Mental Health, Bengbu Medical University, Bengbu, China

**Keywords:** traditional Chinese medicine (TCM), cross-cultural transmission, ethnopharmacology, Yin-Yang, botanical drugs, global healthcare integration

## Abstract

The globalization of Traditional Chinese Medicine (TCM) has facilitated its integration into healthcare systems beyond China, particularly in cross-Asia countries such as Japan, Korea, and Vietnam, while influencing biomedical practices worldwide. This review explores the cultural, historical, and scientific dimensions of TCM’s transmission, focusing on how its foundational theories (e.g., Yin-Yang, Qi-Blood, and Five Elements) and practices (e.g., acupuncture, herbal formulations) have been adapted and validated in diverse sociocultural contexts. We analysed primary literature from data collected by PubMed, Google Scholar, CNKI, Web of Science, Bing, Baidu, and Scopus (1990–2024). Our review critically evaluates the scientific evidence supporting TCM-derived bioactive metabolites like artemisinin from Artemisia annua L. [Asteraceae] and berberine from Coptis chinensis Franch. [Ranunculaceae], examining their concentrations, bioavailability, and clinical applications. Challenges such as standardization, intellectual property disputes, and cultural reinterpretation are critically evaluated. This paper systematically argues that TCM’s cross-cultural transmission reflects a dynamic interplay between tradition and modern biomedicine, offering a model for integrating traditional knowledge into global healthcare while highlighting the limitations of current research and areas requiring further investigation.

## 1 Introduction

Traditional Chinese Medicine (TCM), with its 3,000-year history, represents a holistic medical system rooted in philosophical frameworks such as *Yin-Yang* balance, *Qi-Blood* circumstance, and the *Five Elements* (Wu Xing) (Cheung, 2011; Yao et al., 2013). Over centuries, TCM has spread beyond China through trade routes (e.g., the Silk Road), colonial exchanges, and modern globalization, evolving into hybridized forms such as Japanese Kampo and Korean Hanui (Xiang et al., 2022). This review examines TCM’s transmission to biomedical systems and cross-Asia medical systems, emphasizing its cultural adaptability and potential for scientific validation. As an integrative system combining treatments like acupuncture and botanical drugs, TCM’s pharmacological aspects are inherently intertwined with instrumental therapies, justifying their combined analysis here.

## 2 Methods

We conducted this systematic review according to PRISMA guidelines. From January 1990 to March 2024, we searched four electronic databases (PubMed, Web of Science, CNKI, Bing, Baidu, and Scopus) using the following search terms: “Traditional Chinese Medicine” OR “TCM” OR “Chinese herbal medicine” AND “cross-cultural” OR “transmission” OR “adaptation” OR “integration” OR “globalisation.” Additional manual searches were performed using Google Scholar to identify relevant grey literature.

Inclusion criteria were: (1) original research or review articles published in English or Chinese; (2) studies focusing on the transmission, adaptation, or integration of TCM in countries outside China; (3) articles addressing cultural, historical, or scientific aspects of TCM transmission. Exclusion criteria included: (1) studies focusing solely on clinical efficacy without addressing cultural elements; (2) articles without accessible full text; (3) conference abstracts.

Two independent reviewers screened titles and abstracts, followed by full-text assessment. Data extraction was performed using a standardised form capturing study characteristics, geographical focus, TCM elements discussed, forms of cultural adaptation, and scientific validation evidence. Quality assessment was conducted using the QATQS for observational studies and AMSTAR-2 for reviews. Discrepancies were resolved through discussion with a third reviewer.

## 3 Historical pathways of TCM transmission

### 3.1 Timeline of TCM’s globalization

### 3.2 Spread of TCM

### 3.3 Silk Road herb trade routes

#### 3.3.1 Major traded herbs

***Ginseng quinquefolium (L.) Alph. Wood—*** (China → Middle East/Europe) (Chen et al., 2004).

***Rheum L—*** (Tibet → Persia → Europe for digestion) (Frankopan, 2017).

***Berlinia grandiflora (Vahl) Hutch. & Dalziel—*** (Southeast Asia → India → Mediterranean) (Watson, 1983).

***Lycium barbarum L***. ***—*** Ningxia → Central Asia → Iran → Arabia→ Europe (Jiang et al., 2024).

***Tea—*** (China → Central Asia → Russia via “Tea Horse Road”) (Yang, 2009).

#### 3.3.2 Key routes

**Northern Silk Road**: China → Samarkand → Persia → Rome (for luxury herbs) (Hansen, 2012).

**Maritime Route**: Guangzhou → India → Arabia (e.g., black pepper to China) (Billé et al., 2022).

**Steppe Route**: Mongolia → Russia (e.g., deer antler for *yang* tonics) (Sihui et al., 2010).

### 3.4 Colonial and missionary contributions

European missionaries in the 16th–18th centuries documented TCM practices, translating texts like *Bencao Gangmu* (Compendium of Materia Medica) into Latin, bridging Eastern and Western pharmacopeias (Marcon and Marcon, 2015).

### 3.5 Modern globalization

Post-1970s, TCM gained WHO recognition, with acupuncture adopted in over 180 countries (Zhang et al., 2022). The 2015 Nobel Prize for artemisinin validated TCM-derived drug discovery (Tu, 2011).

## 4 Core cultural elements in TCM’s adaptation

### 4.1 Philosophical frameworks

***Yin-Yang Balance:*** Adapted in Japanese Kampo to diagnose *Kyo-Jitsu* (deficiency-excess) states (Maeda-Minami et al., 2019).

***Qi-Blood circumstance:*** In **TCM**, the concepts of ***Qi*** *-* ***Blood*** and their interaction with **circumstances** (e.g., environment, emotions, lifestyle) form the foundation of diagnosis and treatment (Béres, 2024). This theory is also used to explain metabolic syndromes in Korean (**Korean Oriental Medicine**) (Jeon et al., 2023).

***Five Elements (Wu Xing)***: Korean Hanui links organ networks to emotional health, integrating Confucian ethics (Kim, 2018).

### 4.2 Diagnostic and therapeutic practices

TCM diagnosis, including the pulse diagnosis, tongue diagnosis, channel palpation diagnosis, etc.

***Pulse Diagnosis:*** Modified in Vietnamese medicine as *Mạch học*, emphasizing climatic influences (Phuong et al., 2019).

***Tongue diagnosis***: Tongue diagnosis has gained interest worldwide, particularly in integrative medicine. Tongue diagnosis is part of **Kampo Medicine (Japan)** assessments. Example: **“Juzentaihoto”** (a Kampo formula) prescription may consider tongue coating (Kawanabe et al., 2016). In **Korean Medicine (Hanui),** using **Sasang Constitutional Medicine**, tongue shape helps classify body types (Ko et al., 2013).

***Channel palpation diagnosis***: Japanese **Kampo** practitioners sometimes use **meridian palpation** (経絡触診, *keiraku shokushin*) to assess **“kyo-jitsu” (虚実, deficiency-excess)** patterns (Motoo et al., 2011). Korean medicine uses meridian palpation (**경락 진단, gyeongnak jindan**) alongside pulse diagnosis (**맥진, maekjin**) (Hŏ, 2013).

***Acupuncture*:** European adaptations use electroacupuncture for pain management, diverging from TCM’s meridian theory (MacPherson et al., 2013).

## 5 Scientific validation and pharmacological innovation

### 5.1 Bioactive metabolites

An example of a significant milestone discovery, ***Artemisinin:*** Derived from *Artemisia annua L* (Qinghao), revolutionized malaria treatment, and according to this remarkable scientific achievement, TU YOU YOU was awarded the Nobel Prize in Physiology or Medicine in 2015 (Liu and Liu, 2016; Tu, 2011). The concentration of artemisinin in the plant typically ranges from 0.1%–1.5% dry weight, with bioavailability of approximately 30% when administered orally (Tu, 2011).

***Berberine***: From *Coptis chinensis Franch* (Huanglian), validated for diabetes and hyperlipidemia based on clinical trials showing significant reductions in blood glucose and lipid levels at doses of 0.5–1.5 g daily (Kong et al., 2020). However, bioavailability remains limited (approximately 5%), necessitating higher dosing or innovative delivery systems.

Q**iliqiangxin capsules:** Compositive TCM consists of 11 individual plant metabolites and is used for chronic heart failure with demonstrated efficacy in randomised controlled trials (Li et al., 2013). The standardised preparation contains active markers including astragaloside IV, ginsenoside Rb1, and salvianolic acid B at concentrations of 0.5%, 0.3%, and 1.2%, respectively.

### 5.2 Formulation synergy

***PHY906:*** A Kampo-inspired TCM formula enhancing chemotherapy efficacy (Lam et al., 2010).

### 5.3 Challenges in standardization

***Botanical Drug Variability***: Batch differences in *Salvia miltiorrhiza Bunge* (Danshen) affect clinical outcomes due to variations in tanshinone and salvianolic acid content (Liu et al., 2017). This highlights the critical need for standardisation methods that account for both chemical markers and therapeutic efficacy.

## 6 Case studies of TCM in cross-Asia contexts, cultural reinterpretation and hybridization

### 6.1 Japan: Kampo Medicine

***Integration with Biomedicine***: Kampo is prescribed alongside statins for metabolic syndrome, requiring careful consideration of potential interactions and synergies (Prasad et al., 2020). These integrated approaches necessitate pharmacovigilance and monitoring systems that can detect interactions between botanical and pharmaceutical agents.

### 6.2 Korea: Hanui medicine

***Sa-am Acupuncture:*** Combines TCM with Korean folk traditions, representing a cultural adaptation that maintains core principles while incorporating local medical knowledge (Canaway, 2017).

***Ginseng Cultivation***: *Panax ginseng C.A.Mey* (renshēn) is commercialized as a global adaptogen, with standardised extracts containing specified levels of ginsenosides (typically 4%–8%) (Zahiruddin et al., 2020). This represents both cultural exchange and scientific advancement in standardisation methods.

### 6.3 Vietnam: Southern herbology

***Thuốc Nam:*** Blends TCM with indigenous herbs like *Gynochthodes officinalis (F.C.How) Razafim. & B. Bremer* (Ba Kích) (Zhang et al., 2018).

The term ***Traditional East Asian Medicine (TEAM)*,** coined in the late 20th century, serves as an umbrella term for interrelated medical systems in China (TCM), Japan (Kampo), and Korea (Hanbang) (Hinrichs and Barnes, 2013; Park, 1994). It emphasizes their shared historical foundations and core principles while recognizing regional adaptations and diversity across East Asia (Unschuld, 2003).

## 7 Challenges and controversies

### 7.1 Intellectual property conflicts

***Patent Disputes:*** Japan’s commercialization of *Ephedra sinica Stapf* [Ephedraceae]-based drugs without benefit-sharing represents a significant issue in the global TCM market (Umemura, 2011).

### 7.2 Cultural misappropriation

***Yoga and TCM Hybrids***: Practices labelled as “Zen acupuncture” in some biomedical contexts often dilute TCM’s philosophical depth by removing theoretical frameworks while retaining techniques (Scheid, 2002).

### 7.3 Regulatory hurdles in biomedical healthcare systems (FDA/EMA policies)

The globalization of TCM faces significant regulatory hurdles in Western healthcare systems, primarily due to differing standards for safety, efficacy, and quality control enforced by agencies like the U.S. Food and Drug Administration (FDA) and the European Medicines Agency (EMA) (Dubovitskaya et al., 2019). and also touching on ecological and animal welfare issues (Still, 2003).

## 8 Future directions

***AI-Driven Formulation:*** Machine learning to optimize TCM herb combinations (Misra et al., 2023).

***Global Policy Harmonization***: WHO’s ICD-11 inclusion of TCM categories represents a significant step toward integrating traditional medical knowledge into global healthcare frameworks (Harrison et al., 2021).

***Interdisciplinary Training***–Cross-education for practitioners in both systems could improve mutual understanding.

***Rigorous but Adaptive Study Designs***–Developing better placebo controls and outcome measures for TCM research. The pragmatic scientific approach is not restricted to studies concerned with mechanisms of action.

## 9 Conclusion

**FIGURE 1:**
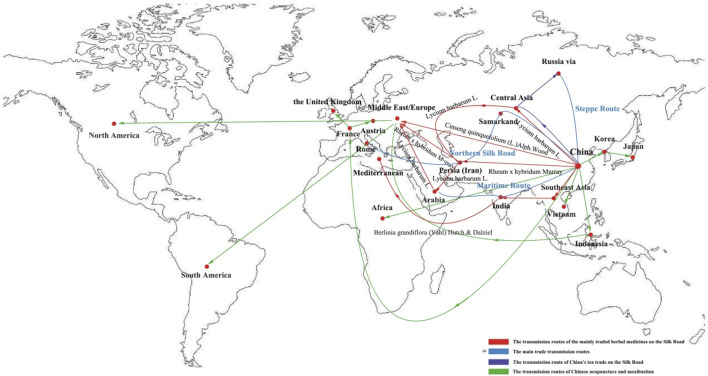
Spread of TCM.

TCM’s cross-cultural transmission exemplifies the dynamic interplay between tradition and globalization (Table 1; Figure 1). While ongoing scientific research, guided by evidence-based medicine, seeks to verify its therapeutic effects, preserving cultural integrity requires balancing standardization with respect for local adaptations. Collaborative frameworks for equitable knowledge-sharing are essential to position TCM as a potential cornerstone of integrative global healthcare, provided that rigorous, culturally sensitive validation processes continue to advance.

**TABLE 1:** Timeline of TCM's Globalization.

Period	Key events
Han Dynasty (206 BCE–220 CE)	The Silk Road trade began, exporting herbs [e.g., *Cinnamomum cassia (L.) J.Presl* and *Glycyrrhiza uralensis Fisch. ex DC*.] to Persia and Rome (Bradley, 2019). Texts such as ***Huangdi Neijing*** influenced Greco-Arabic medicine, evidenced by Ibn Sina’s ***Canon of Medicine*** referencing pulse diagnosis (Winder, 2012)
Tang-Song Dynasty (618–1,271)	TCM texts like ***Shanghan Lun*** spread to Japan/Korea, and Arab traders carried herbs to the Middle East (Liu, 2022). In addition, TCM and other cultures spread to Central Asia through the Sogdi people on the Silk Road (Rong, 2022)
Yuan Dynasty (1,271–1,368)	Mongol Empire integrated TCM with other medicines; rhubarb was traded to Europe (Sivin, 2001)
Ming-Qing (1,368–1912)	Jesuit missionaries documented TCM, tea, and acupuncture, which gained European interest (Anderson, 2017)
20th Century	TCM was banned in China (1920s) but revived post-1949; acupuncture spread globally after Nixon’s 1972 China visit (Anderson, 2017)
21st Century	WHO recognizes TCM (2019); over 100 countries have adopted acupuncture/herbal clinics (Foley, 2025)

The integration of TCM and biomedicine is not merely a technical challenge but a philosophical negotiation. While pragmatic solutions (e.g., combined therapies, AI-assisted diagnostics) are emerging, the deeper question remains: Can two fundamentally different worldviews co-exist in medicine, or must one ultimately subsume the other? Future progress may depend on developing a new meta-framework that respects both systems without forcing either into an alien paradigm.
